# Mapping the single cell spatial immune landscapes of the melanoma microenvironment

**DOI:** 10.1007/s10585-023-10252-4

**Published:** 2024-01-13

**Authors:** Jamie Magrill, Dan Moldoveanu, Jiayao Gu, Mathieu Lajoie, Ian R Watson

**Affiliations:** 1 Rosalind and Morris Goodman Cancer Institute, McGill University, Montréal, QC Canada; 2 Department of Human Genetics, McGill University, Montréal, QC Canada; 3 Department of Biochemistry, McGill University, Montréal, QC Canada; 4 Research Institute of the McGill University Health Centre, Montréal, QC Canada

**Keywords:** Melanoma, Immune checkpoint inhibitors, Tumor microenvironment, Spatial imaging, Spatial transcriptomics, CyTOF-IMC

## Abstract

Melanoma is a highly immunogenic malignancy with an elevated mutational burden, diffuse lymphocytic infiltration, and one of the highest response rates to immune checkpoint inhibitors (ICIs). However, over half of all late-stage patients treated with ICIs will either not respond or develop progressive disease. Spatial imaging technologies are being increasingly used to study the melanoma tumor microenvironment (TME). The goal of such studies is to understand the complex interplay between the stroma, melanoma cells, and immune cell-types as well as their association with treatment response. Investigators seeking a better understanding of the role of cell location within the TME and the importance of spatial expression of biomarkers are increasingly turning to highly multiplexed imaging approaches to more accurately measure immune infiltration as well as to quantify receptor-ligand interactions (such as PD-1 and PD-L1) and cell-cell contacts. CyTOF-IMC (Cytometry by Time of Flight - Imaging Mass Cytometry) has enabled high-dimensional profiling of melanomas, allowing researchers to identify complex cellular subpopulations and immune cell interactions with unprecedented resolution. Other spatial imaging technologies, such as multiplexed immunofluorescence and spatial transcriptomics, have revealed distinct patterns of immune cell infiltration, highlighting the importance of spatial relationships, and their impact in modulating immunotherapy responses. Overall, spatial imaging technologies are just beginning to transform our understanding of melanoma biology, providing new avenues for biomarker discovery and therapeutic development. These technologies hold great promise for advancing personalized medicine to improve patient outcomes in melanoma and other solid malignancies.

## Introduction

Melanoma is an immunogenic malignancy with an elevated tumor mutational burden (TMB) and a highly active immune tumor microenvironment (TME) [1]. While historically associated with poor clinical outcomes, late-stage metastatic melanoma patients have seen a significant improvement in prognosis with the advent and clinical implementation of immune checkpoint inhibitors (ICIs) [2–4]. The favorable response to these therapies has been linked to a number of factors, such as abundance of neoantigens and the degree of infiltrating lymphocytes within the melanoma TME [5–7]. In recent years, spatial imaging technologies have been increasingly used to study the immune infiltration of melanomas, with the goal of understanding the complex interplay between stromal and tumor cells, as well as distinct immune and tumor-infiltrating lymphocyte subsets (TILs), and their effect on ICI response.

Improved approaches to characterize the melanoma TME carries a degree of urgency with clear clinical significance as more than 50% of late-stage metastatic melanoma patients treated with ICIs will either not respond or develop progressive disease [8]. With the recent approval of anti-LAG-3 ICIs for the treatment of metastatic melanoma, a better understanding of the role of the melanoma TME and mechanisms mediating patient response to anti-PD-1 monotherapy compared to combination therapy with anti-CTLA-4 or LAG-3, is clearly needed [9]. Furthermore, results from recent melanoma neoadjuvant clinical trials, SWOG S1801 (NCT03698019) and NADINA (NCT04949113), are poised to redefine management of high-risk resectable stage III melanomas [10]. Molecular profiling studies of pre- and post-treated melanomas in the neoadjuvant setting provides an efficient platform to understand mechanisms of therapy response with measured pathological response available in a relatively short period of time. Such melanoma study designs provide a critical opportunity to identify biomarkers to personalize adjuvant therapy and improve patient outcomes [11]. In this review, we will provide a brief survey of current multiplexed imaging technologies and highlight recent studies that have employed such approaches to characterize the spatial immune landscape of melanomas to better understand ICI responses.

## Multiplexed imaging technologies

Limitations in conventional multiplexed tissue staining and antibody-based imaging, such as hematoxylin and eosin (H&E), immunohistochemistry (IHC) and immunofluorescence (IF) approaches has previously limited the scope and scale of melanoma TME research. However, studies have shown the improved predictive power of biomarkers of ICI response when incorporating spatial TME information, such as cell-type specific expression in specific regions of melanomas [5, 12]. Such studies have reinforced the need to employ more advanced highly multiplexed approaches for translational research.

To address these limitations, various multiplexed spatial imaging methods have been developed to study the melanoma TME in search of new clinically predictive biomarkers over the past decade (Table 1). Multiplexed IF and cyclical multiplexed immunofluorescence techniques such as OPAL-7 [13], tyramide signal amplification (TSA) [14], t-CyCIF (Tissue-based Cyclic Immunofluorescence) [15], or MILAN (Multiple Iterative Labeling by Antibody Neodeposition) [16] can image from 6 to 60 markers using repeated cycles of antibody deposition and imaging. Among these, the more highly-multiplexed technologies have enabled researchers to extract more cell-type specific spatial information, such as t-CyCIF, which makes use of repeated cycles of low-plex fluorescence staining and imaging for up to 60 markers [15]. T-CyCIF has recently been applied to the melanoma TME to evaluate sequential melanoma biopsies obtained from the same patient, revealing co-evolution of differences in immune composition among different clonal lineages, while another study on metastatic melanoma lymph nodes used a 45-plexed t-CyCIF antibody panel to classify prognostically relevant immune-cell neighborhoods [17, 18]. T-CyCIF has also been combined with other multiplexed technologies including PickSeq and NanoString to spatially characterize melanoma cellular neighborhoods, their transition from precursor states to melanoma in situ and invasive tumor, and the immune-suppressive environment along the tumor-stromal boundary [19].

**Table 1:** Imaging methods used to characterize the melanoma spatial microenvironment and example studies

Technology	Imaging Modality	Spatial Resolution	Description	Advantages	Limitations	Reference papers
Multiplexed Immunofluorescence	Immunofluorescence	0.2–0.5 μm	Uses fluorescent probes to label specific targets and visualize them under a microscope.	High spatial resolution, can be used to visualize multiple targets simultaneously, can be used on live cells.	Limited number of markers, potential for photobleaching.	Karras et al., 2022. [20]
OPAL 7-plex Imaging	Immunofluorescence	0.2–0.5 μm	Fluorescence imaging method that allows multiplexed detection of up to six proteins plus DNA in tissue sections.	Lower-cost, multiplexing capabilities and compatibility with standard microscopes.	Limited number of markers, limited to fixed samples and relatively low throughput.	Conway et al., 2022; Nguyen et al., 2021. [13, 21]
Cyclic Immunofluorescence (t-CyCIF)	Immunofluorescence	0.2–0.5 μm	Uses rounds of staining and imaging to generate a multiplexed fluorescence image of tissue sections.	Higher multiplexing capabilities than other immunofluorescence modalities.	Limited to fixed samples and relatively low throughput.	Liu et al., 2021; Maus et al., 2022. [17, 18]
Bright-field Multiplexed Immunohistochemistry	Immunohistochemistry	0.2–0.5 μm	Uses colorimetric labeling to detect multiple antigens (up to three) in tissue sections.	Compatibility with standard microscopes and staining platforms (e.g. Ventana) and some multiplexing capabilities.	Limited number of markers, limited to fixed samples and relatively low throughput.	Ugolini et al., 2022. [22]
Imaging Mass Cytometry (CyTOF-IMC)	Mass Cytometry	1 μm	Uses antibodies conjugated to metal ions to label targets and mass spectrometry to detect them.	High throughput, can analyze many targets simultaneously (up to 40 markers).	Requires specialized equipment and specialized expertise for analysis.	Harris et al., 2022; Hoch et al., 2022; Martinez-Morilla et al., 2021; Moldoveanu et al., 2022. [23–26]
Matrix-assisted Laser Desorption/ionization (MALDI)	Mass Spectrometry	5 μm	Uses matrix-assisted laser desorption/ionization to generate a mass spectrometry signal that can be used to image specific molecules or detect molecular signals within tissue sections.	High throughput, can analyze many analytes simultaneously.	Requires specialized equipment and specialized expertise for analysis.	Casadonte and Caprioli, 2011; Casadonte et al., 2021. [27, 28]
Multiplexed ion beam imaging (MIBI-TOF)	Time-of-flight Secondary Ion Mass Spectrometry	~ 0.25 μm	Uses a beam of ions to image tissue and generate a mass spectrometry signal that can be used to image multiple targets at high resolution.	High spatial resolution, can analyze many targets simultaneously (up to 40 markers), allows rescanning of slides at multiple resolutions.	Requires specialized equipment and specialized expertise for analysis.	Taylor et al., 2021. [29]
Spatial transcriptomics	RNA barcoding with UV-photocleavable linkers	10 μm (for GeoMx), 50 nm (for CosMx)	Captures gene expression information in situ via RNA sequencing of tissue sections. Wide selection of assays including NanoString GeoMx (employing NGS-based Digital Spatial Profiling) and NanoString CosMx (employing Cyclic in-Situ Hybridization).	High-throughput, can provide information on gene expression patterns within tissues.	Limited to transcriptomics data, GeoMx provides high-throughput whole transcriptome information at the cellular-compartment level through fluorescence markers, does not provide information on protein localization. CosMx provides single-cell information on a more limited panel of RNA and proteins.	Hoefsmit et al., 2020; Thrane et al., 2018. [30, 31]
Visium	Oligo capture probes	55 μm	Molecular profiling of mRNA by spatially barcoded mRNA-binding oligonucleotides.	High-throughput and spatially resolved transcriptome data.	Limited resolution compared to other forms of spatial transcriptomics, analyzes ‘patches’ of 5–50 cells.	Quek et al., 2021. [32]

Recent advancements in lower-cost spatial immune profiling technology include bright-field multiplexed imaging, which enables imaging of triple-plexed bright-field colors for clinical and histopathological diagnostic assays using standard pathology-lab equipment [22]. Another advancement is nine-color multiplexed immunofluorescence imaging that has been applied to characterize cell phenotype diversity and immunosuppression patterns within the TME of malignant pleural mesothelioma [33].

In contrast, mass-spectroscopy-based imaging and analysis methods, such as MALDI (Matrix Assisted Laser Desorption/Ionization), DESI (Desorption Electrospray Ionization), SIMS (Secondary Ion Mass Spectrometry), and MIBI-TOF (Multiplexed Ion Beam Imaging by Time-of-Flight MS), can detect up to thousands of analytes including metabolites on a sample without using repetitive imaging cycles [29, 34, 35]. For example, MIBI-TOF, which can image up to 36-plex markers (or in theory, up to 100 markers) and uses a tunable ion-beam that can be adjusted to various sample depths and image resolutions, has been applied to image cellular phenotypes and tissue structure, revealing regional variability in tumor cell phenotypes in several different breast cancer subtypes including triple-negative breast cancer [35, 36]. CyTOF-IMC, another recent addition to the spatial imaging toolkit, can detect up to 40 proteins on a single sample of formalin-fixed paraffin-embedded (FFPE) tissue and has recently been applied to characterize the melanoma TME [23–26].

Exponential development in the spatial transcriptomics imaging field has enabled researchers to spatially image transcripts from up to 20,000 different protein-coding genes at mid to high-resolution, using technologies including NanoString [37], Visium [38], and MER-FISH [39]. Generally, spatial transcriptomics technologies fall into two categories. First, those that spatially segment tissue into a limited number of relevant cell types and interrogate large numbers of transcripts across those selected groups of cells, albeit without achieving single-cell resolution, such as NanoString [37, 40]. Second, those that provide single-cell, or close-to single-cell resolution for a more limited number of transcripts, such as MER-FISH [40]. In the intermediary zone are technologies that include Visium, which allow for lower-resolution whole transcriptome sequencing of ‘patches’ of 5–50 cells [38, 41]. These technologies have provided detailed insights into the TME in the context of ICI treatment for melanoma liver metastases. A comprehensive analysis of cutaneous and uveal melanoma liver metastases identified differences in the TME between these melanoma subtypes, such as PD-L1 expression and the ratio of exhausted CD8 T cells to several other T cell subsets [30]. High-plex spatial RNA profiling has been instrumental in revealing cell type-specific biomarker expression in the TME during early melanoma evolution, including prominent expression of S100A8 and S100A9 in melanoma-adjacent keratinocytes [42]. Furthermore, these technologies have been used to examine the immune landscape of tumor-associated lymph nodes in melanoma, leading to the identification of potential biomarkers of CD11c activation that could be clinically relevant for predicting survival outcomes [37].

Spatial transcriptomics platforms, including NanoString and Visium, have greatly advanced our understanding of gene transcription by facilitating the detailed analysis of mRNA levels at a high spatial resolution [37–41]. Of note, transcriptomic-based technologies do possess inherent limitations as some isoforms may not be accurately differentiated by these platforms and mRNA levels are not always congruent with protein expression. Thus, protein-based methodologies cannot be wholly substituted by spatial transcriptomics alone. Integration of spatial proteomic and transcriptomic profiling allowed by currently available platforms has already shown promise in offering a more holistic view of the cellular landscape [23, 24]. However, the field still faces significant challenges in merging these data streams, largely due to technological limitations in comprehensively measuring both the transcriptome and proteome simultaneously.

## CyTOF studies in cutaneous melanoma

Flow cytometry has long been a cornerstone in the field of cellular analytics, providing a means to measure, quantify and analyze characteristics of single cells in suspension in a multiplexed workflow. The Helios CyTOF platform introduced the use of rare earth metal isotopes for antibody labeling and integrated mass spectroscopy into flow cytometry as an analysis technique, thus allowing for separation of cells based on their mass-to-charge ratio and eliminating issues with spectral overlap from traditional flow cytometry platforms [43, 44]. This significantly expanded the capabilities of flow cytometry technology and allowed for the simultaneous analysis of up to 60 parameters using metal-tagged antibodies, albeit without preserving spatial orientation of cells within tissues [44–46]. The technology used to develop the Helios CyTOF platform paved the way for the development of the Hyperion CyTOF-IMC platform that can multiplex image up to 40 markers using rare earth metal isotope-conjugated antibodies [47].

**Fig. 1:**
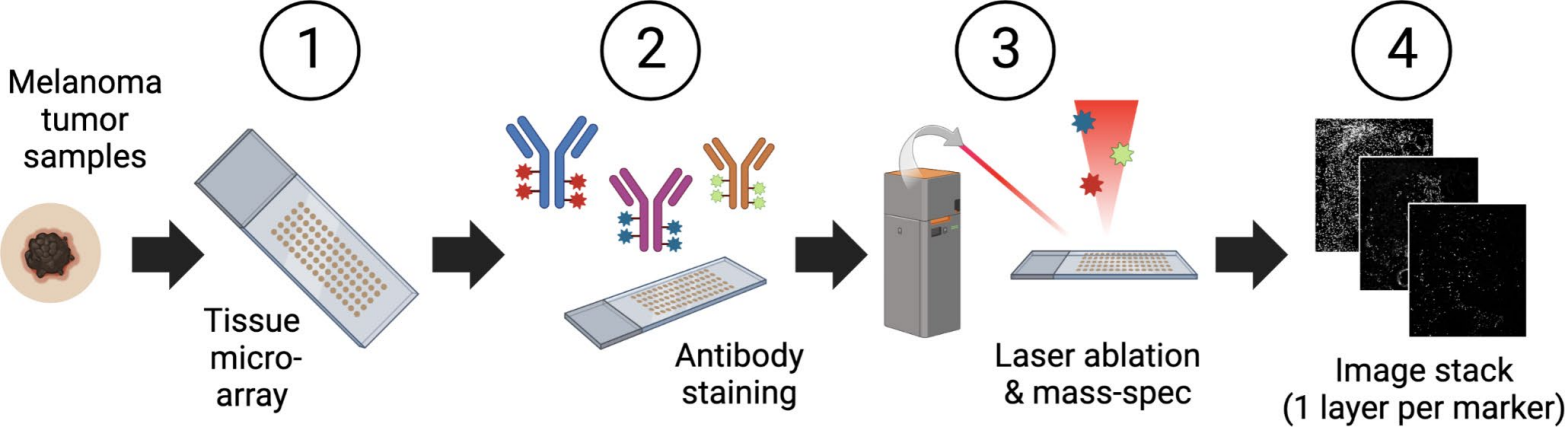
Illustration of the data acquisition workflow used for CyTOF-IMC: (1) One ~ 5 μm-thick section of a tissue microarray (TMA), usually composed of 1 mm2 melanoma cores (2) is stained with a cocktail of antibodies labeled with metal isotopes (colored asterisks). (3) Samples are ablated with a high energy laser in a rastered pattern, and the resulting ionized isotope plumes are analyzed by a mass cytometer, which returns the number and type of metal isotopes per pixel (one pixel = 1micron x 1micron). (4) Each antibody results in a single image per sample, and together are layered to construct a multi-image stack. Since 2014, CyTOF-IMC has been utilized to spatially analyze a wide array of tissues and tumor-types, including breast, lung, and melanoma, as well as to identify biomarkers of response to treatment in breast cancer (including HR and HER2) or immunotherapy (anti PD-1 or anti-CTLA4) in melanoma [24, 26, 47, 60, 61]

CyTOF-IMC can detect antibodies at sub-cellular resolution simultaneously on the same tissue slide using high-frequency laser ablation of antibody-tagged metal isotopes in a low-dispersion laser ablation chamber, for either FFPE or fresh tissue [47]. In brief, tissues are labeled with metal-tagged antibodies using standard immunohistochemistry methods, air dried, laser-ablated pixel by pixel, and transported by a mixed argon and helium stream to the CyTOF mass cytometer for measurement (Fig. 1) [47]. Cellular images generated by CyTOF-IMC undergo a two-step analytical process. Images are first segmented into individual cells using a variety of software. Next, cells are phenotyped using methods based either on unsupervised clustering (e.g. Phenograph) [48], FlowSOM [49], or probabilistic classification models (e.g. Astir) [50] (Table 2 provides a non-exhaustive overview of some of the more commonly-used tools available for these tasks). Advanced spatial analysis, including the calculation of median intercellular distances, and contact enrichment, can be performed using tools such as imcRtools [51] (see [52] for a more comprehensive review of analytical pipelines).

**Table 2:** Summary of different segmentation and classification tools for CyTOF-IMC data

Segmentation Tools					
Tool Name	Architecture or Methods	Shared Characteristics	Notable Features of Each Tool	Implementation languages	Reference Papers
Cellpose	Convolutional Neural Network	Little to no parameter tuning. Requires no annotation to start. Application to a wide range of cell images. Allow users to re-train the model.	Allows users to correct labels and retrain the model in one user interface.	Python	Stringer et al., 2020. [53]
StarDist	Convolutional Neural Network		Integrated in many image annotation tools for easy label generation.	Python	Schmidt et al., 2017. [54]
DeepCell (Mesmer)	Convolutional Neural Network producing cell boundaries and cell masks		Insensitive to low-resolution images.	Python	Greenwald et al., 2021. [55]
IMC segment-ation pipeline (Ilastik + CellProfiler)	Random Forest classifier classifies pixels into nuclei vs. background vs. cytoplasmic pixels (ilastik). Watershed and other object segmentation techniques (CellProfiler).	Requires parameter tuning for optimal results.	Customizable modular pipelines. Allow users to correct labels and retrain model. User-friendly for biologists.	Python	Zanotelli and Bodenmiller, 2022. [56]
CIRCLE	Convolutional Neural Network combined with traditional segmentation techniques.		Requires no annotations to start.	MATLAB	Karimi et al., 2022. [57]
Classification Tools					
Tool Name	Method	Shared Characteristics	Notable Features of Each Tool	Implementation languages	Reference Papers
FlowSOM	Self-organizing map (SOM) combined with consensus clustering.	Unsupervised clustering followed by manual annotation of cluster phenotypes. Can identify novel cell populations. Requires parameter tuning for optimal results.	Good visualization aid.	R	Gassen et al., 2015. [49]
flowMeans	K-means clustering followed by a merging procedure.		Stable as the size of dataset increases.	R	Aghaeepour et al., 2010. [58]
Phenograph	Uses Louvain algorithm to identify communities in a KNN graph.		Good for capturing inner structure of the data.	MATLAB/Python	Levine et al., 2015. [48]
Astir	Variational inference to model marker distributions using user-defined cell-type marker lists.	Classification based on user-provided definition of cell phenotypes. Designed for highly multiplexed imaging technology.	Requires prior knowledge of cell-type specific markers.	Python	Geuenich et al., 2021. [50]
CELESTA	Score-based cell type assignment that uses spatial information.		Requires parameter tuning for rare cell types.	R	Zhang et al., 2022. [59]

Two of the earliest publications to use CyTOF to characterize melanoma patient samples utilized the Helios platform to characterize peripheral blood mononuclear cells (PBMCs) or immune profiles of tumor biopsies from patients treated with anti-PD-1 monotherapy, or combined anti-PD-1 and anti-CTLA-4 [62, 63]. One early study conducted on stage IV melanoma patients receiving anti-PD-1 immunotherapy found that the frequency of CD14 + CD16 − HLA-DRhi monocytes in PBMCs prior to therapy initiation was a strong indicator of progression-free and overall survival, suggesting their potential use in informing treatment decisions [62]. Another study identified activated T-cell signatures and T-cell populations in responders to both treatment modalities (anti-PD-1 and anti-CTLA-4) using a panel of multiplexed antibodies to characterize immune cell populations [63]. Further analysis revealed an EOMES + CD69 + CD45RO + effector memory T-cell phenotype that was significantly more abundant in responders to combined immunotherapy than non-responders. The gene expression profile of this population was associated with longer progression-free survival in patients treated with single-agent therapy and showed greater tumor shrinkage in both treatments, revealing insights into response and resistance mechanisms to ICIs [63]. These early CyTOF flow cytometry studies provided valuable information regarding abundance of immune cell populations present in the melanoma TME. However, the advent of the Hyperion IMC has facilitated the multiplexed characterization of spatial relationships of cells and biomarkers within the melanoma TME using the CyTOF platform.

Four recent studies have utilized CyTOF-IMC to directly characterize the melanoma TME (Table 3). Published in 2021, CyTOF-IMC was employed to analyze the TME of patients with metastatic melanoma who received ICI, and to identify indicative factors of treatment response [25]. Rather than segmenting cells, the authors used a newly designed version of the AQUA software to measure marker intensity in molecularly defined compartments. Multivariable analyses revealed significant associations of 12 markers with progression-free survival, and 7 markers with overall survival, which included b2-microglobulin [25].

**Table 3:** Studies using CyTOF-IMC to characterize the melanoma TME, including a brief summary and sample sizes

Title	Authors	Year Published	Brief Summary	Sample Size
Biomarker Discovery in Patients with Immunotherapy-Treated Melanoma with Imaging Mass Cytometry	Martinez-Morilla S, et al. [25]	2021	This study used CyTOF-IMC to identify biomarkers of treatment response in patients with metastatic melanoma who received immunotherapy, using a newly designed version of the AQUA software for image analysis to measure 25 markers on FFPE samples, identifying 12 markers for progression-free survival and 7 markers for overall survival, including beta2-microglobulin.	Multiple cohorts, including a Discovery cohort: 60 pretreatment samples from metastatic melanoma patients treated with immune checkpoint inhibitors, a Prognostic Cohort: samples from 131 historic untreated melanoma patients, and a Validation cohort: samples from 121 advanced melanoma patients who received PD-1 blockade treatment
Spatially mapping the immune landscape of melanoma using imaging mass cytometry	Moldoveanu D, et al. [26]	2022	This study used CyTOF-IMC to quantify the expression of 35 protein markers and identify melanoma, lymphocyte subsets, macrophage/monocyte, and stromal cell populations, revealing that the relative abundance of proliferating antigen-experienced cytotoxic T cells, and their proximity to melanoma cells are associated with a positive response to immune checkpoint inhibitors.	5 benign nevi and 67 melanomas
Enriched circulating and tumor-resident TGF-β(+) regulatory B cells in patients with melanoma promote FOXP3(+) Tregs	Harris RJ, et al. [23]	2022	This study used a panel of 34 CyTOF-IMC antibodies and other multiplexed methods to investigate the role of B-cells in anti-tumor adaptive immune responses in melanoma patients, and revealed the presence of regulatory cytokine-expressing B-cell populations and their interactions with T-cells and T-regulatory cells, which may contribute to immunosuppression in the tumor microenvironment.	26 melanoma patients and 12 age-matched healthy volunteers
Multiplexed imaging mass cytometry of the chemokine milieus in melanoma characterizes features of the response to immunotherapy	Hoch T, et al. [24]	2022	This study used multiplexed mass cytometry-based imaging of protein markers and RNA transcripts, to characterize the chemokine landscape and immune infiltration in metastatic melanoma samples, identifying a correlation between CXCL9 and CXCL10 expression and dysfunctional T cells, as well as the potential role of T-cells in B-cell recruitment and B-cell follicle formation in tumors with B cells.	Samples from 69 patients with metastatic melanoma

In our study, published in 2022, we utilized CyTOF-IMC to profile more than 230,000 individual cells from 5 benign nevi and 67 melanomas and identified melanoma, lymphocyte, macrophage/monocyte, and stromal cell populations, allowing for in-depth spatial quantification of the melanoma microenvironment [26]. While prior studies have shown that the abundance of TILs in the TME is associated with better prognosis and response to ICIs [64–66], we demonstrated that within the pre-treatment melanoma TME, it was the abundance of proliferating antigen-experienced cytotoxic T-cells (CD8 + CD45RO + Ki67+) and their proximity to melanoma cells that best informed on ICI responses in our analyzed cohort [26]. This indicated that a shorter distance between melanoma and nearby antigen-experienced cytotoxic T-cells within the TME was linked to a favourable response, highlighting the potential of CyTOF-IMC to quantify spatial cell-cell interactions for ICI biomarker studies.

Two other studies were published in 2022 that utilized CyTOF-IMC but combined this approach with other modalities to investigate the immune environment of melanoma. Specifically, researchers investigated the role of regulatory and pro-inflammatory cytokine-expressing B cells in patients with melanoma, utilizing flow cytometry, CyTOF-IMC, single-cell RNAseq, immunofluorescence staining and transcriptomic analysis [23]. The group found that patients had enhanced circulating regulatory B cell populations and reduced pro-inflammatory B cell populations compared to healthy volunteers [23]. They also found that cytokine-expressing B cells in the melanoma TME assembled in clusters and interacted with T-cells and T-regulatory cells via various signaling pathways [23]. Patient-derived B cells were found to promote T-regulatory cell differentiation and T-helper cell proliferation in a TGF-β-dependent manner, an effect further enhanced with anti-PD-1 checkpoint blockade [23]. These findings highlight the bidirectional crosstalk between B and T cell subsets with immunosuppressive attributes in the context of melanoma. In another CyTOF IMC study, researchers reported the use of multiplexed mass cytometry–based imaging of protein markers and RNA transcripts to investigate the chemokine landscape and immune infiltration in metastatic melanoma samples [24]. The study showed that tumors lacking immune infiltration had low levels of antigen presentation and markers of inflammation, and were devoid of most of the profiled chemokines [24]. In contrast, infiltrated tumors expressed high cytokine levels, with CXCL9 and CXCL10 being localized in patches associated with dysfunctional T-cells expressing the B lymphocyte chemoattractant CXCL13 [24]. This study also found that T-cells play a role in B cell recruitment and potentially in B cell follicle formation, and that the formation of tertiary lymphoid structures may be accompanied by naïve and naïve-like T-cell recruitment, which can contribute to antitumor activity. These two CyTOF-IMC articles demonstrate the potential of combining multiple spatial ‘omic’ technologies to make important TME discoveries.

Future studies combining multiple spatial ‘omic’ technologies (e.g. CyTOF-IMC, NanoString, Visium, ATAC-Seq, microbiome analysis, and plasma metabolite analysis) will undoubtedly lead to a more deeper understanding of TME biology. One challenge with integrating multiple profiling approaches is that often these technologies require different biological material inputs (i.e. plasma, tissue sections, or tissue lysate). Already, studies have expanded IMC technologies to detect mRNA and proteins with single-cell and spatial resolution in melanoma and breast tumours, capturing three mRNAs along with a panel of proteins to reveal correlations between mRNA and proteins at both the single-cell and cell population levels [24, 67]. Furthermore, we recently interrogated microbiome composition, plasma metabolite makeup, and employed CyTOF-IMC to analyze the immune landscape of melanoma tumors prior to fecal microbiota transplantation (FMT) in order to more comprehensively characterize the immune infiltrates in responsive and non-responsive patients to combination FMT and ICI therapy [68].

## Limitations

Spatial imaging proteomic and transcriptomics technologies, while powerful, are newer techniques that often grapple with limitations and compromises. These include balancing single-cell resolution with high multiplexing capabilities, limitations in availability of required reagents such as high-quality antibodies and probes, as well as challenges associated with high costs and slow data acquisition times coupled with the complexity of data analysis. For example, analyzing and interpreting the large and complex datasets produced by CyTOF-IMC requires sophisticated tools and computational expertise. Recent advances in machine learning have allowed for improved cell segmentation and classification in the analysis and interpretation of large and complex datasets produced by CyTOF-IMC. For example, a recent study utilized CyTOF-IMC and deep learning to analyze the TME of lung adenocarcinoma samples from 416 patients, profiling 1.6 million cells using a combination of classical and modern machine learning based computer vision algorithms in order to reveal distinct immune lineages and activation states with clinical correlates, and demonstrating the potential of artificial intelligence in predicting patient progression using a single 1-mm^2^ tumor core [57, 61]. Finally, as the technology for spatial imaging and transcriptomics continue to advance, analytical tools, imaging analysis methods and data processing pipelines will need to keep pace. These rapid advances provide both great opportunities and present unique challenges to researchers, as consolidation of best-practices for application and data analysis are continually evolving to meet the needs of the ever-advancing armamentarium of spatial imaging technologies.

## Conclusion

The future of single-cell spatial profiling will be driven by the development of novel technologies that can overcome current limitations and provide more nuanced insights into single-cell dynamics. These advancements will enable multiplexed, cell-type specific, and tumor location-specific analysis of biomarkers, which could significantly enhance their predictive power. Furthermore, the integration of 3D spatial imaging using technologies such as CyTOF-IMC may provide a more comprehensive view of the cellular landscape. Ultimately, the integration of 3D biomarker imaging across multi-omic platforms would significantly improve our understanding of the TME [69].

Within the melanoma TME research field, these single cell imaging advancements are poised to have an immediate and profound impact. With the recent approval of anti-LAG-3 for the treatment of late-stage metastatic melanoma, identification of biomarkers that can inform on prescription of monotherapy anti-PD-1, versus combinations that include anti-CTLA-4 and LAG-3, are urgently needed. Moreover, single cell technologies amenable to predicting response in the neoadjuvant setting could significantly advance personalized medicine by tailoring adjuvant treatments to individual patient profiles [11].

As our review has underscored, recent studies utilizing single cell imaging approaches are just beginning to illuminate the cellular composition and functional dynamics of the melanoma TME. This area of investigation has the potential to uncover novel biomarkers for diagnosis and prognosis, particularly in the context of ICI response, and to identify new targets for therapeutic intervention.

## Search methodology

In this review, a search of peer-reviewed scientific articles and literature reviews was completed using PubMed with the following search terms: (“Melanoma”) AND (“Tumor micro-environment” OR “Tumour micro-environment” OR “Tumor microenvironment” OR “Tumour microenvironment” OR “Cellular Microenvironment” OR “Cellular Micro-environment” OR “Tumour immune microenvironment” OR “Tumor immune microenvironment” OR “Tumour immune micro-environment” OR “Tumor immune micro-environment”) AND (“spatial landscape” OR “spatial”), within the date range of 2014–2023 (the earliest CyTOF-IMC paper being released in 2014), retrieving 82 results. After full-text review, we included 3 papers on melanoma spatial imaging using CyTOF-IMC, and captured an additional 6 papers on other melanoma spatial imaging technologies. We also applied a snowballing search methodology using the references cited in the articles identified in the literature search, locating an additional 1 pertinent paper (see Table 3). For the snowballing search, articles were limited to those including CyTOF-IMC or other relevant spatial imaging technologies, melanoma or another relevant cancer, or other pertinent articles. Each identified item was assessed for relevance by a member of the study team and final calls were made by the senior author. This review is intended to summarize high-impact, relevant and recent literature on spatial imaging technologies, specifically CyTOF-IMC, for the tumor microenvironment and immune landscape in melanoma, and is not meant to be exhaustive.
